# The Case for Optimized Edge-Centric Tractography at Scale

**DOI:** 10.3389/fninf.2022.752471

**Published:** 2022-05-16

**Authors:** Joseph Y. Moon, Pratik Mukherjee, Ravi K. Madduri, Amy J. Markowitz, Lanya T. Cai, Eva M. Palacios, Geoffrey T. Manley, Peer-Timo Bremer

**Affiliations:** ^1^Lawrence Livermore National Laboratory, Livermore, CA, United States; ^2^Department of Radiology and Biomedical Imaging, University of California, San Francisco, San Francisco, CA, United States; ^3^Argonne National Laboratory, Lemont, IL, United States

**Keywords:** connectomes, identifiability, tractography, diffusion MRI, optimization, EDI, edge-centric

## Abstract

The anatomic validity of structural connectomes remains a significant uncertainty in neuroimaging. Edge-centric tractography reconstructs streamlines in bundles between each pair of cortical or subcortical regions. Although edge bundles provides a stronger anatomic embedding than traditional connectomes, calculating them for each region-pair requires exponentially greater computation. We observe that major speedup can be achieved by reducing the number of streamlines used by probabilistic tractography algorithms. To ensure this does not degrade connectome quality, we calculate the identifiability of edge-centric connectomes between test and re-test sessions as a proxy for information content. We find that running PROBTRACKX2 with as few as 1 streamline per voxel per region-pair has no significant impact on identifiability. Variation in identifiability caused by streamline count is overshadowed by variation due to subject demographics. This finding even holds true in an entirely different tractography algorithm using MRTrix. Incidentally, we observe that Jaccard similarity is more effective than Pearson correlation in calculating identifiability for our subject population.

## 1. Introduction

The structural connectome is a powerful framework for analyzing macro-scale circuity of the living human brain and associating this connectivity with behavioral traits and health outcomes. Streamlines (also called fiber tracks or samples) are computationally reconstructed from each seed voxel in the white-to-gray matter boundary and connect exactly two regions of the brain. Structural connectome analysis, or connectomics, may have the power to distinguish autism spectrum disorder, estimate patient age and gender, and even predict cognitive ability (Betzel et al., 2014; Ingalhalikar et al., 2014; Contreras et al., 2015; Roine et al., 2015). Furthermore, there is a significant expectation that connectomics will provide crucial insights into otherwise difficult-to-probe neurological conditions, such as traumatic brain injury (TBI) and other cognitive disorders.

However, the anatomic validity of connectomes based on diffusion MRI has been inconsistent (Maier-Hein et al., 2017; Jeurissen et al., 2019). Tractography algorithms based on local fiber orientation may reconstruct large numbers of erroneous streamlines without additional constraints from ground-truth observation. Furthermore, the reconstructed streamline density may differ greatly from actual streamline density at each voxel, even when adjusted with filtering techniques such as SIFT (Smith et al., 2014, 2015). Owen et al. (2015) propose edge density imaging (EDI), which maps the number of region-to-region edges that pass through every white matter voxel. EDI is generated by edge-centric tractography, which reconstructs streamlines as edge bundles between individual pairs of cortical and subcortical regions. Each edge bundle is confined to its own anatomically-plausible volume, which helps to exclude invalid streamlines. This has the advantage of normalizing connections between regions and improving inter-subject reproducibility, particularly between regions with high edge density (Owen et al., 2016).

However, progress in EDI and edge-centric tractography has been hampered by the computational cost of generating an order of magnitude more streamlines than before. A traditional connectome will simply seed a specific quantity of streamlines per voxel in the white-to-gray matter boundary and determine in which region each streamline terminates. This can be accomplished with a few tens of millions of streamlines and may take at most a few hours on modern computers. Edge-centric tractography must be repeated for each region-pair, such that each voxel (in the white-to-gray matter boundary) will reconstruct streamlines for every single region-pair that its streamlines could possibly intersect. Even when excluding anatomically-implausible region-pairs, this process can easily require billions of streamlines and consume many nodes on the most advanced high performance computing (HPC) platforms. Creating and curating edge-centric connectomes for a few dozen patients, even at a research facility, may take weeks and requires dedicated personnel familiar with computational neuroscience. As a result, processing hundreds or thousands of patients for a large-scale study has been cost-prohibitive. Here, we exploit the Department of Energy's vast HPC capabilities to examine the probabilistic variation of edge-centric tractography and the predictability of its computations. This aspect of EDI has not been studied carefully because of the sheer scale of computational and human resources required to analyze a large number of connectomes.

In particular, we focus on the probabilistic algorithms underpinning edge-centric tractography, particularly PROBTRACKX2 (Behrens et al., 2003) and MRTrix (Tournier et al., 2019). Though our thesis only applies to probabilistic algorithms, we include the results of a deterministic algorithm from MRTrix to demonstrate the robustness of identifiability as a quality metric. To account for the potential of crossing tracks, imprecise white-to-gray matter boundary estimations, and uncertainty induced by the lack of spatial resolution in the MRI scans, the research standard has been to compute 1,000 streamlines per voxel per region-pair (Owen et al., 2015). The unofficial publication standard is as many as 5,000 streamlines. However, the advantage of 1,000 streamlines per voxel remains unclear and the results presented below suggest that there may be little practical benefit in computing more than 1 streamline per voxel per region-pair. This simple but significant change implies an immediate reduction in computational cost by up to three orders of magnitude without significant loss of information.

The tractability of structural connectomes to matrix analysis has resulted in a variety of proposed techniques to assess their reliability (Imms et al., 2019). But since most of these techniques target specific medical or anatomical conditions, it is difficult to use them as universal metrics. In this work, we utilize a more general notion of identifiability, introduced by Amico and Goñi (2018). Conceptually, identifiability measures how well one can identify the connectome of a specific patient among a cohort of participants given an independently computed connectome from a prior MRI scan. Identifiability provides a generic measure of the information content of structural connectomes that is independent of any particular health condition or metric. We use a multi-center cohort of participants admitted for orthopedic, i.e., non-head related, injuries in order to demonstrate that a large streamline count does not improve identifiability in a general population. More specifically, we find that connectomes computed using 1 streamline per voxel per region-pair are as descriptive as connectomes that were generated with significantly higher streamline counts. Furthermore, the random variance induced by the probabilistic tractography is often as big as any changes observed for higher streamline counts. These two facts combined imply that many standard analyses will perform just as well with connectomes generated from a small number of streamline count than what is currently considered the standard. Reducing streamline count drastically reduces the computational resources required, making edge-centric structural connectomes accessible to a much wider range of researchers and potentially paving the way for real-time connectome analysis in a clinical setting.

## 2. Methods

The edge-centric tractography workflow consists of three major steps (Payabvash et al., 2019): (1) calculating the probability distributions of fibers within each voxel from the raw MRI data, (2) parcellating the brain into structurally relevant regions, and (3) estimating how strongly each pair of regions are connected. The main focus of this paper is to analyze heuristics for the connectivity between brain regions using different streamline counts and use that information to estimate the accuracy of different levels of optimization. These heuristics must, in essence, estimate the likelihood that reconstructed connectomes match the real-world connectome. Since computing this likelihood directly is challenging, the accepted approach is to use uniform random sampling. Specifically, we begin with a large number of streamlines at each seed voxel and subsequently approximate the likelihood values by dividing the number of successful streamlines by the total number of streamlines. The likelihood values are then normalized by the volume of the regions and inserted into the connectome. Each cell of this upper-triangular matrix represents the connectivity of a region-to-region pair.

When we increase the streamline count, this process will converge to the true connectome as defined by the given parcellation, local fiber directions, and tractography algorithm. As the fiber directions form a very high dimensional sampling space and a complex distribution, common wisdom would suggest that a very large number of streamlines are required for an accurate estimate. The exact origin of the accepted publication standard of streamlines, between 1,000 and 5,000 streamlines per voxel. remains unclear. But these numbers are likely the result of similar concerns regarding accuracy. However, while more streamlines undoubtedly add more information to the connectome, doing so repeatedly for every single region-pair generates enormous amounts of redundant data. If we use 82 cortical and subcortical regions in the commonly-used Desikan-Killiany parcellation, this results in 6642 potential region-pairs. Even when we curate the number of plausible region-pairs in the same way as Payabvash et al. (2019), we have nearly 1,000 region-pairs to consider for each seed voxel. Given between 10,000 and 100,000 seed voxels in the white-to-gray matter boundary (depending on subject anatomy, image resolution, and voxel density), this can result 10 to 100 billion streamline computations. We contend that this is far in excess of requirements for most use cases.

It is well-known that the physical aspects associated with an MRI procedure, i.e., measurement noise, patient motion, etc., as well as the constant change of the human brain add significant uncertainties to the measurements made on the brain which affect the generated connectome (Burgess et al., 2016). Therefore, it is unproductive to compute the connectome to a precision that is significantly higher than the maximal resolution implied by the inherent uncertainties. However, quantitatively assessing the “quality” of a connectome is not straight forward. There are two significant challenges. The first challenge is the requirement of a sufficient number of comparable MRI scans and the resources to compute their corresponding connectomes at different streamline counts. The second challenge is that there is no agreed-upon comparison metric between connectomes to understand the level of differences relevant in practice.

Here we address the first problem through a collaboration with the Transforming Research and Clinical Knowledge in Traumatic Brain Injury (TRACK-TBI) consortium.^1^ TRACK-TBI is a longitudinal, observational study of TBI carried out at 18 Level 1 Trauma Centers across the United States. It includes brain-injured subjects along with a matched cohort of orthopedic injury control subjects. All participants were followed for 12 months following injury, and MRIs were collected from a subset of both the brain-injured and orthopedic injury cohorts. To avoid potential bias from the actual brain injuries, we are using a cohort of 88 orthopedic injury control subjects all between ages 18 and 71 (mean 37.8 yr; SD 13.7 yr; 30 female). All patients have no indication of head trauma based on clinical screening. We utilize diffusion-weighted MR imaging for each patient at two time points: 2 weeks and 6 months after injury. MR imaging is conducted with 3T scanners at 11 sites across the United States. All images are acquired using a uniform single-shell sampling scheme. All sites use the same acquisition parameters, insofar as possible across GE, Philips, and Siemens platforms (Palacios et al., 2017). Diffusion MRI and T1-weighted MRI pre-processing and post-processing are as reported in Owen et al. (2015, 2016). This process ultimately provides NIfTI diffusion tensor images with b = 1,000 s/mm^2^, divided into 2.7-mm isotropic voxels in a 128 L × 128 W × 72 H matrix.

Given a total of 176 MRI scans we utilize MaPPeRTrac (Moon et al., 2020), a new portable and parallel computing pipeline that enables us to exploit large-scale computing facilities for the necessary tractography computations.^2^ Our pipeline accomplishes the tractography workflow using the software components shown in Table 1.

**Table 1:** Software components of MaPPeRTrac.

**Pre-processing**	**BET, DTIFIT, FLIRT (Jenkinson et al., 2002)**
Segmentation	Freesurfer (Desikan et al., 2006)
Fiber tensor estimation	BEDPOSTX2 (Behrens et al., 2003)
Probabilistic tractography	PROBTRACKX2 (Behrens et al., 2003)
Alternative prob. and deterministic tractography	MRTrix3 (Tournier et al., 2019)

**Figure 1:**
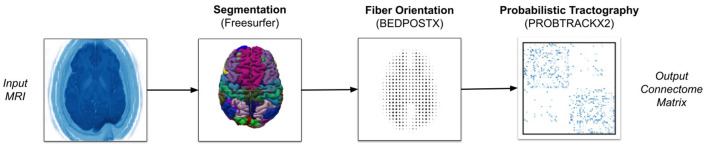
Overview of MaPPeRTrac pipeline.

Figure 1 gives a rough illustration of how we convert NIfTI images to connectomes matrices. When running Freesurfer, we parcellate the brain with the Desikan-Killiany atlas. For the PROBTRACKX2 pipeline, we use BEDPOSTX2 to estimate fiber orientation directions (FOD). We then run PROBTRACKX2 for each region-pair while adjusting streamline count between 1 and 1,000 streamlines per voxel and using the gray-white matter boundary as the seeding volume. All other software components are left to their default values. Our tractography workflow is portable across most scientific HPC clusters with Slurm, Cobalt, or Grid Engine job scheduling. However, to process these particular subjects, we used machines running the TOSS 3 operating system with Slurm scheduling. Further details can be found in Table 2 (Moon et al., 2020).

**Table 2:** Hardware used to run MaPPeRTrac.

**System**	**CPU**	**Clock speed**	**Cores/**	**RAM/**	**GPU**
			**node**	**node**	
Quartz	Intel Xeon E5-2695	2.10–3.30 GHz	36	128 GB	n/a
Pascal	Intel Xeon E5-2695	2.10–3.30 GHz	36	256 GB	NVIDIA Tesla P100

**Figure 2:**
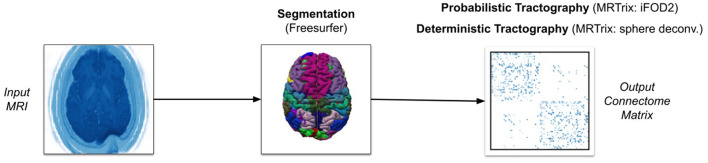
Overview of alternative pipeline with MRTrix.

Our software can also conduct tractography using the MRTrix library, as shown in Figure 2. It is important to note that we ran a traditional tractography algorithm using MRTrix. Since MRTrix lacks the ability to track the number of streamlines passing through each voxel, as opposed to just the start and end regions, it cannot be used to generate EDI. Our main intention with MRTrix is to show the generalizability of the claim that extremely high streamline counts fail to provide unique information, regardless of algorithm details and parameters. We conducted these experiments with the same of number of streamlines as edge-centric tractography to demonstrate this point.

Our MRTrix pipeline uses the same pre-processing tools and Freesurfer parcellation as the PROBTRACKX2 pipeline (Tournier et al., 2007). However, we convert the parcellation to five-tissue-type (5TT) format in order to use the Anatomically-Constrained Tractography (ACT) framework (Smith et al., 2012). This framework will more accurately terminate streamlines. We then estimate the response function for each white-matter voxel using the (Tournier et al., 2013) iterative algorithm, since this is the recommended approach for single-shell data. Having specified a mask using the whole diffusion-weighted image, we run the spherical deconvolution algorithm proposed by Tournier et al. (2007) on the response function estimation to generate the FOD. After normalizing the FOD to correct for intensity outliers, we use this FOD as the input for either the iFOD2 algorithm for probabilistic tractography or the SD_STREAM algorithm for deterministic tractography. The iFOD2 algorithm conducts second-order integration of estimated fiber orientations to determine principle streamline direction (Tournier et al., 2010). The SD_STREAM algorithm performs Newton optimization to orient streamlines toward local peaks in the fiber orientation (Tournier et al., 2012). Like with PROBTRACKX2, we seed streamlines at the center of each voxel in the gray-white matter boundary and adjust streamline count between 1 and 1,000 streamlines per voxel. But whereas our PROBTRACKX2 pipeline seeded only the starting region in each region-pair, our MRTrix pipeline must combine all gray-white matter boundary volumes to create a single seeding volume. Since masking was performed during spherical deconvolution on our FOD, we do not apply another mask during tractography. Unless previously indicated, all MRTrix parameters are left to their default values. The hardware and subject data are identical to those used with PROBTRACKX2.

Our goal is to optimize tractography such that computation is minimized without losing any information content. Information content in this context refers to any biomarkers extrapolated from the connectome which may relate to various psychiatric disorders. These biomarkers are essentially patterns in the connectome matrix which are valuable insofar as they can be associated with patient outcomes, such as depressive disorder or Alzheimer's disease. However, despite significant advances, most studies of structural connectomes in a clinical context remain limited to a small number of patients. As a result, it is difficult to point to any set of best practices for tractography optimization in studies with dozens or hundreds of patients.

As previously mentioned, we use the notion of identifiability introduced by Amico and Goñi (2018). Whereas they measured identifiability in functional connectomes, we extend the concept to structural connectomes in order to estimate the information content across different streamline counts. Identifiability assumes that the connectome must capture unique characteristics of the individual, or at least distinct enough to make accurate medical and/or scientific predictions. Given the evidence for this assertion (Finn et al., 2015), we should be able to identify individual patients within a cohort of similar patients as long as each patient's unique characteristics are borne out in their connectome. Identifiability formalizes this concept and provides a quantitative measure of how well we can identify connectomes.


(1) $$\begin{array}{r} A_{ij}=corr\left(p_{i},q_{j}\right) \end{array}$$

(2) $$I_{self}=\frac{1}{N}\sum^{\text{​}}A_{ii}\;and\;I_{others}=\frac{1}{N^{2}-N}\sum^{i\neq 1}\;A_{ij}$$

(3) $$\begin{array}{r} I_{diff}=\left(I_{self}-I_{others}\right)*100 \end{array}$$

The identifiability score for each patient is computed by comparing their connectome at one timepoint *p* to every connectome generated at different timepoints, *q*. As discussed in more detail below we have experimented with various forms of connectome metrics such as correlation, L2 distance, and Jaccard similarity. Equation (1) shows that this results in an *N* × *N* matrix **A**, composed of correlations between the two timepoints where *N* is the number of patients. The average of diagonal elements, *I*_*self*_, measures correlation between connectomes of the same patient. The average of off-diagonals, *I*_*others*_, measures correlation between connectomes of different patients. These can be expressed as in Equation (2). Identifiability *I*_*diff*_, as seen in Equation (3), is measured as the difference between *I*_*self*_ and *I*_*others*_.

Amico and Goñi (2018) improve identifiability by reducing connectome dimensionality. If we perform principal component analysis (PCA) reconstruction with *m* components, then the best possible identifiability we can extract from the data is


(4) $$\begin{array}{r} I_{diff}*=arg\;\underset{m\epsilon M}{\max}\;I_{diff}\left(m\right) \end{array}$$

We express identifiability as Equation (4) in all subsequent sections, as it represents the strongest identification ability for any set of connectomes.

Identifiability can be used to compare the success of different procedures at preserving the connectomes' information content. However, larger study populations will necessarily have lower identifiability, since each patient must self-identify out of a wider pool of candidates. To mitigate this, we calculate the mean identifiability of repeated k-fold validation with fixed-size subsets. We randomly select a subset of *k* patients out of *n* total population, calculate identifiability of the subset, repeat this *r* times, and average the repetitions. The resulting mean identifiability enables comparison between differently-sized populations.


(5) $$\begin{array}{r} A_{ij}=\frac{\left|p_{i}-q_{j}\right|}{\left|p_{i}|+|q_{j}\right|} \end{array}$$

(6) $$\begin{array}{r} A_{ij}=\frac{p_{i}}{\left|p_{i}\right|}\cdot \frac{q_{j}}{\left|q_{j}\right|} \end{array}$$

(7) $$\begin{array}{c} A_{ij}=\frac{\sum_{k}min(p_{ik},q_{jk})}{\sum_{k}max(p_{ik},q_{jk})} \end{array}$$

It is possible to calculate identifiability using correlation metrics other than Pearson correlation. The comparison between test and retest connectomes (see Equation 1) can be expressed using any linear correlation algorithm. For example, Equation (5) demonstrates a comparison using L2 distance, normalized against each connectome. We also examine the normalized dot product (Equation 6) and the Jaccard similarity coefficient (Equation 7). We experiment with multiple correlation metrics to help demonstrate the robustness of our optimization argument.

## 3. Results

We re-ran probabilistic tractography with the same MRI scans for twenty iterations: at five streamline counts with four samples, each initialized with different random seeds. Note that we do not present median or standard deviation for these figures—this is because the cost of computation is so high that generating more than four samples per streamline count would be prohibitive. The five streamline counts are 10, 50, 200, 500, and 1,000 streamlines per voxel per region-pair. In the following figures, each data point represents the mean identifiability at a particular streamline count and random seed. Our tractography workflow re-calculates streamlines for every region pair, so each white matter voxel at the gray-white matter boundary will actually originate many more streamlines than this number suggests. We do not observe a relationship between mean identifiability and streamline count, especially considering stochastic variation and the narrow Y-axis. Since identifiability is the total percentage difference in correlation between *I*_*self*_ and *I*_*others*_ (see Equation 3), small stochastic variations of fractions of a percent have little impact. However, even stochastic variation appears to have a greater impact than streamline count. This suggests that connectomes generated with low streamline counts contain just as much information as high streamline counts, at least for identification tasks.

**Figure 3:**
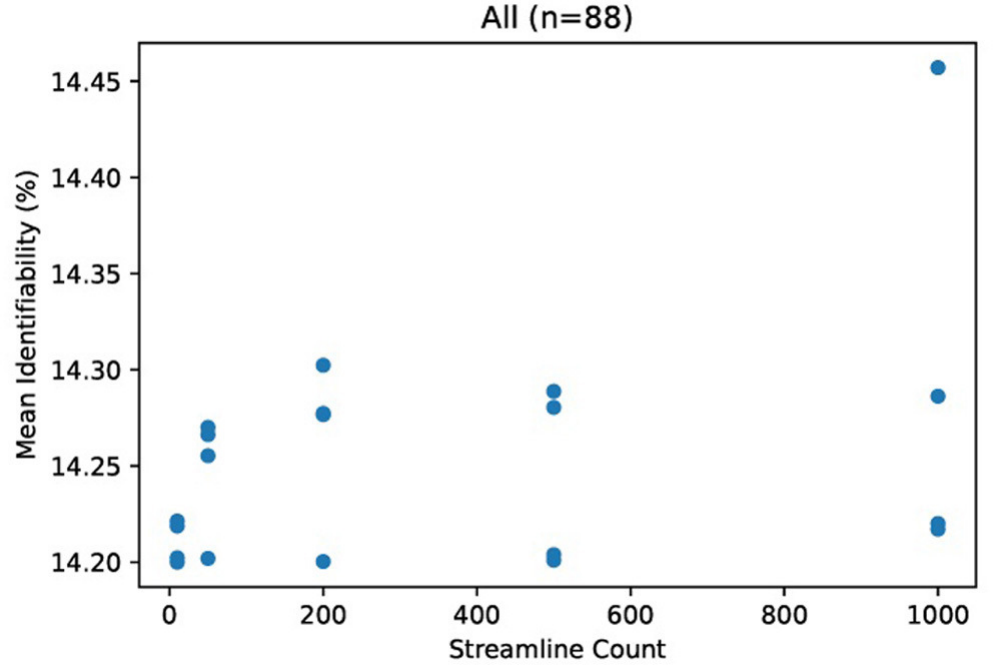
Mean identifiability with all patients (*q* = 4 random seeds per streamline count, *k* = 20 subset size, *r* = 10 repetitions). For each streamline count, there are q data points with tractography running a unique random seed. Each data point represents the average of r repetitions of k randomly selected patients in order to normalize for dataset size.

**Figure 4:**
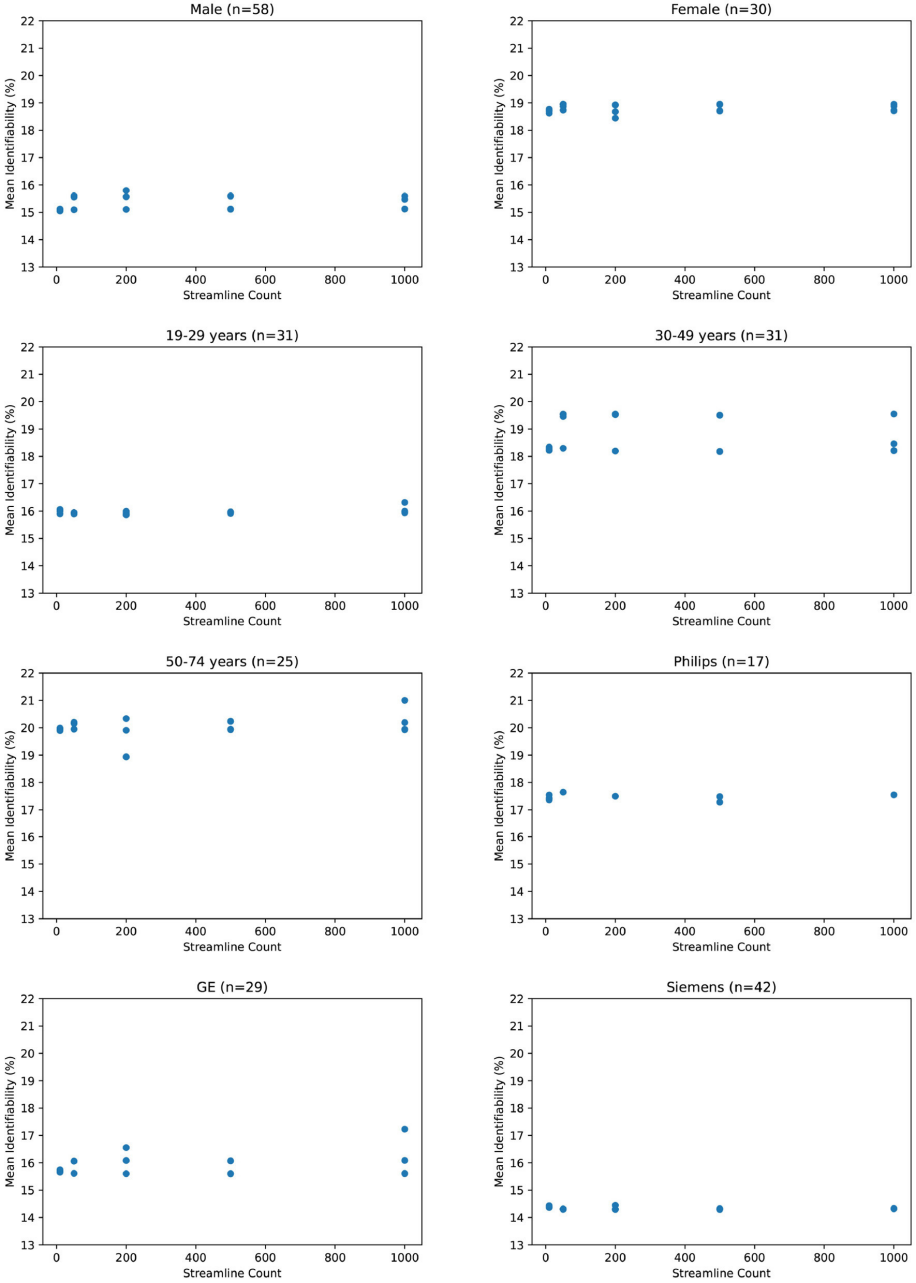
Mean identifiability by category (*q* = 4 random seeds per streamline count, *k* = 20 subset size, *r* = 10 repetitions).

Due to the small number of data points (related to the extreme cost of compute), it would be unhelpful to evaluate correlation metrics between streamline count and identifiability such as coefficient of determination or error bars. We do not deny that correlation may exist between identifiability and streamline count. Because we argue that this correlation is not significant compared to variations due to demographics, we instead consider the absolute variations of identifiability within a category and between categories. In Figure 3, we see variation within all subjects of just 0.25 percent. In comparison, most categories in Figure 4 differ from each other by much greater than 1 percent.

If we zoom in to individual categories, we see that mean identifiability does not strongly vary with streamline count no matter how patients are grouped together. Variation within each category is an average of 0.6 percent. The greatest variation is within 50–74 year olds at 2.1 percent, but this variation shows no positive relationship streamline count and identifiability. In addition, we observe that certain categories present stronger differences than others. Male and female identifiability differ by 3.9 percent, the youngest and oldest patients by 4.1 percent on average, and various MRI platforms by less than 1 percent. Though this does not confirm that identifiability is reading population differences between categories, it does suggest that those differences would be more significant than any increase in identifiability from a higher streamline count.

**Figure 5:**
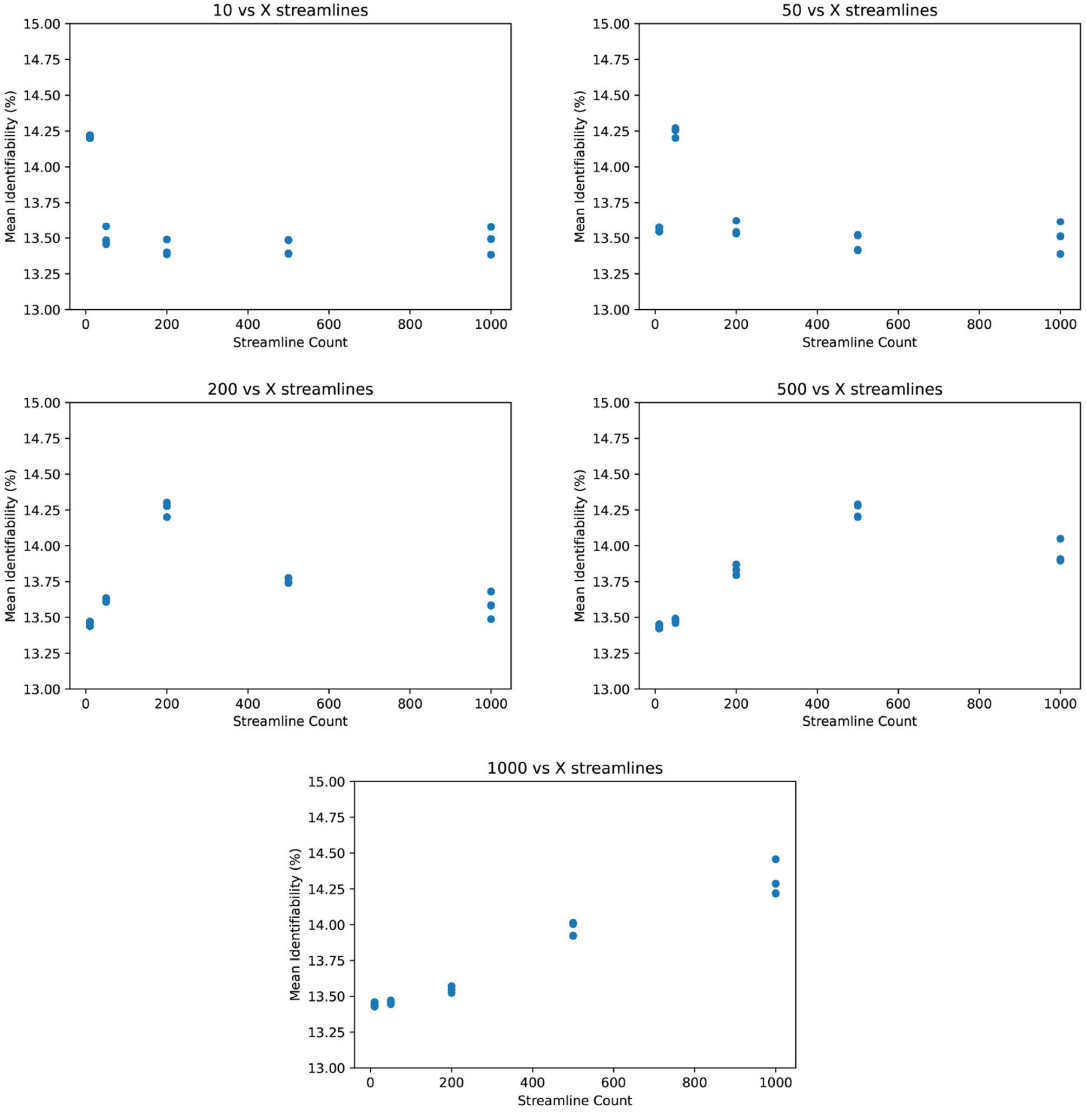
Mean identifiability across streamline counts (*q* = 4 random seeds per streamline count, *k* = 20 subset size, *r* = 10 repetitions).

One could argue that by comparing connectomes only against other connectomes at the same streamline count, identifiability is biased by processing artifacts unique to that streamline count. Considering this, we compared identifiability with test connectomes *p*_*i*_ against retest connectomes *q*_*j*_ from different streamline counts. Figure 5 appears to confirm this bias because identifiability is higher when the test and retest share the same streamline count. But to some degree, this is expected, as information particular to that streamline count is shared between its tests and retests, whereas those from different streamline counts may not carry that information. Nevertheless, the degree of bias does not seem to be significant compared to the overall success in identification. Again note the narrow Y-axis - even identifiability as low as 13% is more than sufficient to distinguish a retest from all 87 other retest connectomes.

**Figure 6:**
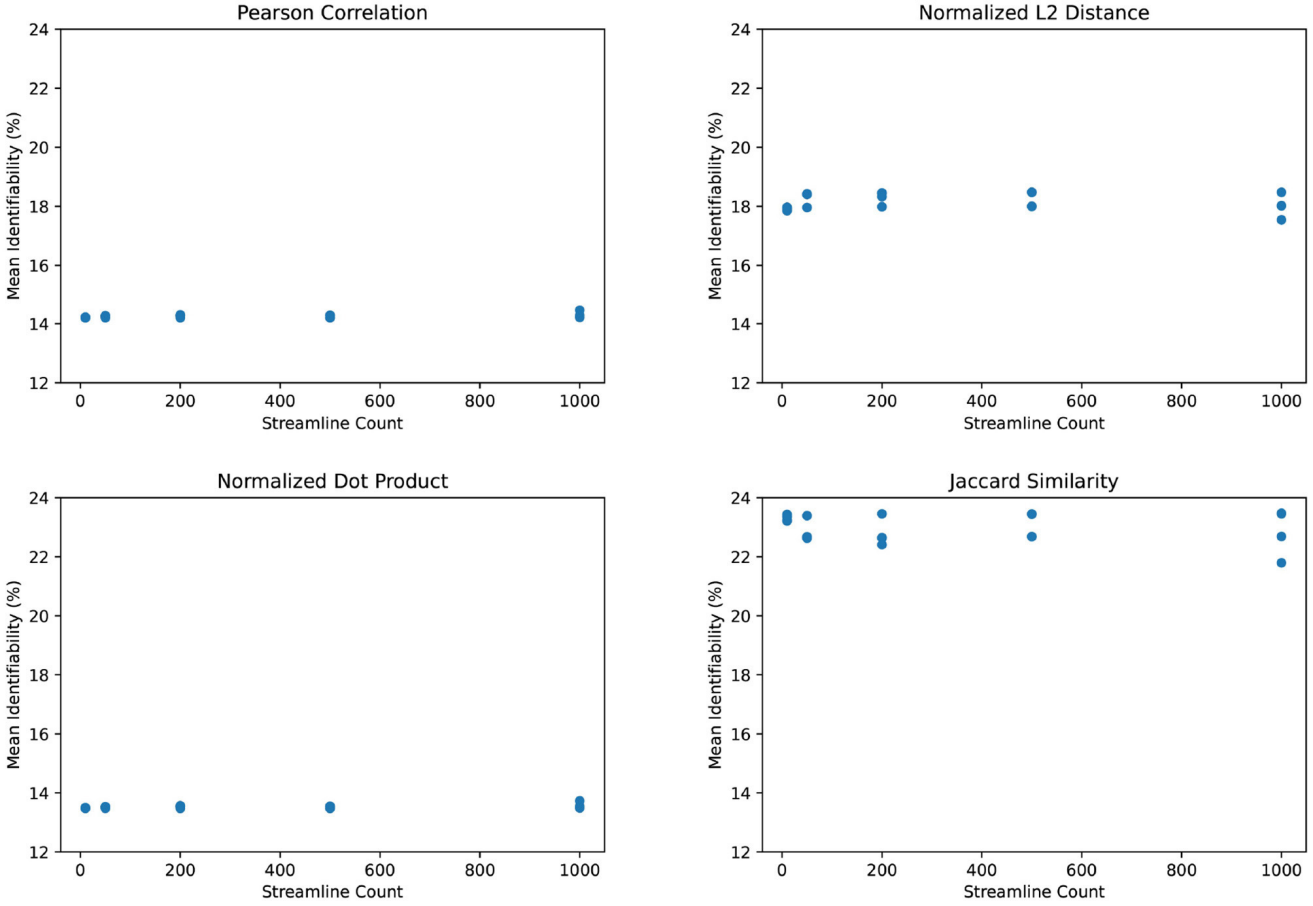
Mean identifiability by correlation metric (*q* = 4 random seeds per streamline count, *k* = 20 subset size, *r* = 10 repetitions).

We observe the same trend of weak correlation between streamline count and identifiability in Figure 6. Incidentally, we find that L2 distance yields somewhat better identification power than Pearson correlation. The normalized dot product appears relatively weak in comparison. However, the Jaccard similarity coefficient demonstrates significantly stronger identifiability than Pearson correlation. This is particularly unusual since Jaccard similarity discards much information from its inputs by only selecting the maximum and minimum of the test and retest values. Although we use Pearson correlation in all other figures due to its prevalence in existing literature, Figure 6 suggests that there may be room for improving the identifiability algorithm.

**Figure 7:**
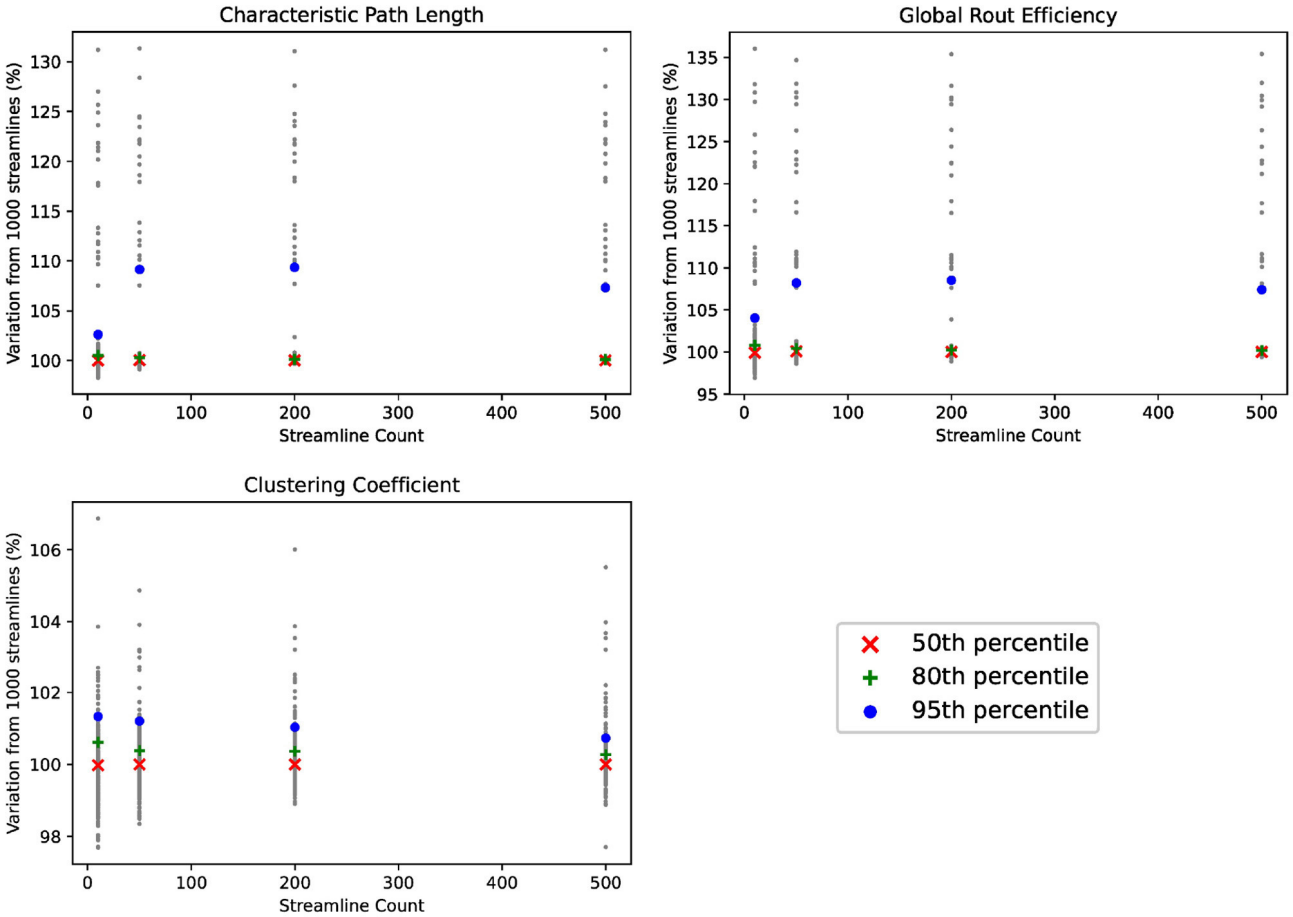
Comparison of graph metrics.

For sake of completeness, we examine the edge-centric connectomes using alternative graph metrics common in neuroimaging literature. Details of these graph metrics for the purpose of investigating test-retest reliability have been described by Imms et al. (2019). For each connectome, we (1) calculate each graph metric at each streamline count, (2) normalize the graph metric at each streamline count against the value of the graph metric at 1,000 streamlines, and (3) plot each normalized graph metric in Figure 7. The resulting plots demonstrate no added value above 1 streamlines per voxel per region-pair, similar to our results for identifiability.

**Figure 8:**
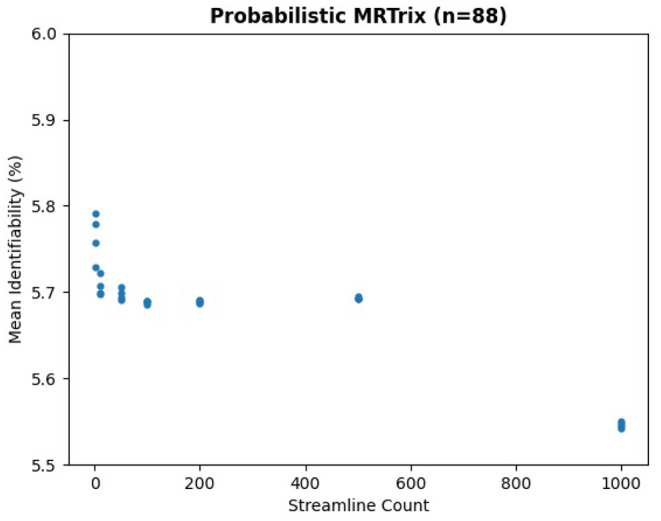
Mean identifiability with all patients using probabilistic MRTrix (*q* = 4 random seeds per streamline count, *k* = 20 subset size, *r* = 10 repetitions, iFOD2 algorithm).

**Figure 9:**
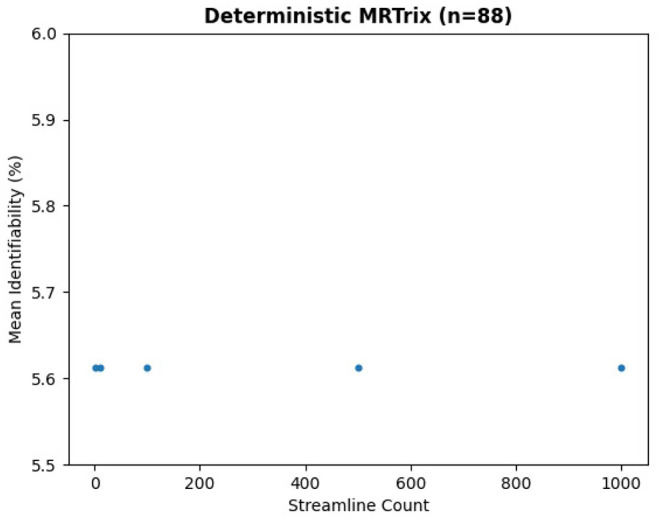
Mean identifiability with all patients using deterministic MRTrix (SD_STREAM algorithm).

We ran the same subjects with MRTrix to generate traditional connectomes, again using five streamline counts with four samples each and k-fold validation. The results in Figure 8 demonstrate the same trend—an extremely slight variation in identifiability with streamline count. In fact, the relationship between streamline count and identifiability appears so tenuous that higher counts have slightly lower identifiability. In Figure 9, it is unsurprising to see the deterministic algorithm sees no variation with streamline count at all. This indicates that the deterministic algorithm used by MRTrix is conducting needless computation beyond the first streamline per voxel, since there is no remaining decision space for tractography to explore.

## 4. Discussion

By comparing edge-centric connectomes with the concept of identifiability, we find that probabilistic and deterministic algorithms do not significantly benefit from high streamline counts. This has major ramifications for the computational cost and availability of edge-centric tractography, as similar results can be achieved with a fraction of the streamlines. However, there is a major risk that optimization would lose information not captured by identifiability. The ability to identify a patient is necessary to connectome analysis—otherwise one could argue that a connectome is indistinguishable and therefore dominated by noise and external variables. But even if we could perfectly identify patients from connectomes, this may not be sufficient for more complex analyses.

There is also the risk that we did not compute sufficient samples. To address this, we re-ran probabilistic tractography on all patients with five streamline counts and four different random seeds, for a total of twenty iterations. With that amount of data, streamline count does not appear to significantly influence identifiability. Even if correlation can be established, the slope of such a curve is so flat as to be swamped by noise and subject demographics. However, it is remotely possible that running far more than twenty iterations would show strong variation. We do not pursue this possibility owing to the computational expense of tractography with high streamline counts - generating our data already consumed over 300,000 CPU hours.

We also find that Jaccard similarity outperforms more commonly used connectome correlation metrics such as Pearson correlation in the calculation of identifiability. Though we are surprised that this is the case, it is possible that Jaccard similarity increases the weight of low-frequency information by effectively binarizing the non-shared values. When calculating identifiability, high-frequency values, such as dense contiguous sections of the brain, may often match to the wrong subject. Subjects are better distinguished by low-frequency areas with unique structures. Given an incorrect match, choosing a minimum or maximum of the test and retest value in low-frequency areas will create a strongly fluctuating test-retest variation since values tend not to overlap. And whereas Pearson correlation and other metrics would dilute this variation by the weight of high-frequency areas, Jaccard similarity would provide consistent test-retest variation in high-frequency areas since it does not combine the test and retest in each voxel. As a result, Jaccard similarity improves identifiability similarly to PCA reconstruction, by pruning low-information data. However, this is mostly speculation and would require further study beyond the scope of this paper.

There is also the concern that our findings lack external physiological data. Brains do not exist in a vacuum, so key markers such as clinical survey results, blood pressure, and body weight may influence connectome analysis in subtle ways. We mitigate this to an extent by categorizing patients by age and gender and find that nothing in these categories undermines our argument regarding streamline count. Furthermore, it has been demonstrated that tractography is highly sensitive to choice of processing method. If the method itself diverges from ground truth, there is little that reproducibility can do to recover accurate results. Ideally, we would approach ground truths using phantom studies on the MRI processing techniques (Nath et al., 2020) or histological studies on *ex vivo* specimins (Schilling et al., 2018, 2019). However, we do not possess further anatomical or physiological data for this patient population, so the influence of other external variables remains unexplored.

We are also limited to using a particular set of acquisition and pre-processing parameters. Previous studies have used a broad array of parameters on the same subjects to make generalizable observations (Côté et al., 2013). Though our narrow parameters may appear to limit the generalizability of our findings, we contend that differences between scans of the patients are subtle enough that the ability of distinguish between them is more significant than the ability to compare alternative parameters on the same data. For example, a slightly different parcellation would result in changes to the overall structure of the connectome matrix, but identifiability would not greatly change since the relative differences between connectomes would be much less affected. Since we can even find the same results with two entirely different tractography softwares, PROBTRACKX2 and MRTrix, then minor changes on tractography parameters are unlikely to change our overall findings.

## 5. Conclusions

Progress in EDI connectomics has been limited by the steep computational cost of probabilistic white matter fiber tractography. Creating diverse datasets with large numbers of patients requires optimizations of the tractography workflow. However, excessive optimization may degrade the connectome's information content. To measure the extent to which we can optimize tractography, we use identifiability as an approximate measure of the average information content in a set of connectomes. Identifiability is a quantifiable metric for identification tasks predictiveness using a patient's test and retest, based on MRI conducted 6 months apart. This enables us to optimize computation by determining whether information is lost.

Edge-density probabilistic tractography is computationally expensive because it simulates massive quantities of white-matter fiber streamlines. We find that the number of streamlines can be greatly reduced from current practice. This optimization appears to have no impact on identifiability; ergo, it does not degrade the connectome's information content for most purposes. Reducing the number of streamlines yields direct linear efficiencies, such that using half the streamlines takes approximately half the time to compute. Existing literature uses between 1,000 and 5,000 streamlines per voxel per region-pair to ensure a well-converged solution. We find that identifiability is stable with as few as 1 streamlines per voxel per region-pair.

We find that low streamline counts perform just as well as high streamline counts even when analyzing our study population with different demographics. These findings hold true for male and female patients, different age ranges, different correlation metrics, and all three common MRI hardware platforms. The choice of population makes a far greater impact than any decision on streamline count. In fact, variations in mean identifiability due to streamline count are even less than those from stochastic variation due to probabilistic tractography.

Using low streamline counts promises to greatly accelerate study of EDI and edge-centric connectomes. High streamline counts do not appear to harm identifiability in any scenario, and will likely continue to be the standard for small-scale studies. But by reducing the computational cost of tractography, this simple optimization will enable hundreds to thousands of edge-centric connectomes to be generated on systems that previously handled a few dozen. Many open neuroimaging questions related to EDI cannot be answered with small-scale studies alone, particularly those on subtle population differences such as behavioral disorders. As the field of connectomics grows, optimizations such as these will be necessary to keep up with the large amount of clinical data and computational resources applied to human brain research as well as foster clinical applications that require faster results for real-time patient care.

## Data Availability Statement

The raw data supporting the conclusions of this article will be made available by the authors, without undue reservation.

## Ethics Statement

Ethical review and approval was not required for the study on human participants in accordance with the local legislation and institutional requirements. The patients/participants provided their written informed consent to participate in this study.

## Author Contributions

Code, experimentation, and writing were primarily conducted by JM under the supervision of P-TB. RM and LC contributed significant technical and editorial collaboration, particularly with the Mappertrac software. The patient data was prepared by the UCSF authors, led by GM. PM and EP contributed most of the Section 2, with significant assistance from AM. All authors assisted with the creation of this paper. All authors contributed to the article and approved the submitted version.

## Funding

The research was funded by the United States Department of Energy under the DOE Office of Science, Advanced Scientific Computing Research. Support was organized under The Co-Design for Artificial Intelligence and Computing at Scale for Extremely Large, Complex Datasets projects (Grant #KJ040301). This document was prepared as an account of work sponsored by an agency of the United States government. Neither the United States government nor Lawrence Livermore National Security, LLC, nor any of their employees makes any warranty, expressed or implied, or assumes any legal liability or responsibility for the accuracy, completeness, or usefulness of any information, apparatus, product, or process disclosed, or represents that its use would not infringe privately owned rights. Reference herein to any specific commercial product, process, or service by trade name, trademark, manufacturer, or otherwise does not necessarily constitute or imply its endorsement, recommendation, or favoring by the United States government or Lawrence Livermore National Security, LLC. The views and opinions of authors expressed herein do not necessarily state or reflect those of the United States government or Lawrence Livermore National Security, LLC, and shall not be used for advertising or product endorsement purposes.

## Conflict of Interest

GM discloses grants from the United States Department of Defense—TBI Endpoints Development Initiative (Grant #W81XWH-14-2-0176), TRACK-TBI Precision Medicine (Grant #W81XWH-18-2-0042), and TRACK-TBI NETWORK (Grant #W81XWH-15-9-0001); NIH-NINDS—TRACK-TBI (Grant #U01NS086090); and the National Football League (NFL) Scientific Advisory Board—TRACK-TBI LONGITUDINAL. The United States Department of Energy supports GM for a precision medicine collaboration. One Mind has provided funding for TRACK-TBI patients stipends and support to clinical sites. He has received an unrestricted gift from the NFL to the UCSF Foundation to support research efforts of the TRACK-TBI NETWORK. He has also received funding from NeuroTruama Sciences LLC to support TRACK-TBI data curation efforts. Additionally, Abbott Laboratories has provided funding for add-in TRACK-TBI clinical studies. AM receives funding from the Department of Defense TBI Endpoints Development Initiative (Grant #W81XWH-14-2-0176) and TRACK-TBI NETWORK (Grant #W81XWH-15-9-0001). She also receives salary support from the United States Department of Energy precision medicine collaboration and the philanthropic organization, One Mind. JM and P-TB are employed by Lawrence Livermore National Security, LLC. The remaining authors declare that the research was conducted in the absence of any commercial or financial relationships that could be construed as a potential conflict of interest.

## Publisher's Note

All claims expressed in this article are solely those of the authors and do not necessarily represent those of their affiliated organizations, or those of the publisher, the editors and the reviewers. Any product that may be evaluated in this article, or claim that may be made by its manufacturer, is not guaranteed or endorsed by the publisher.

## Footnotes

^1^ https://tracktbi.ucsf.edu

^2^ https://github.com/LLNL/MaPPeRTrac
